# Compartment syndrome in infants and toddlers

**DOI:** 10.1007/s11832-016-0766-0

**Published:** 2016-08-18

**Authors:** Alexander Broom, Mathew D. Schur, Alexandre Arkader, John Flynn, Alex Gornitzky, Paul D. Choi

**Affiliations:** 1 Children’s Orthopaedic Center, Children’s Hospital Los Angeles, 4650 Sunset Boulevard, Mailstop #69, Los Angeles, CA 90027 USA; 2 Department of Orthopaedic Surgery, Children’s Hospital of Philadelphia, Philadelphia, USA

**Keywords:** Compartment syndrome, Fasciotomy, Infection, IV infiltration, Pediatric

## Abstract

**Purpose:**

To study the cause, diagnosis, treatment and outcome of acute compartment syndrome in infants and toddlers aged <3 years.

**Methods:**

Fifteen patients aged <3 years with acute compartment syndrome were identified from two large pediatric trauma centers over a fifteen-year period. All children underwent fasciotomy. The mechanism of injury, time of injury, time to diagnosis, compartment pressures, time to fasciotomy, and outcome at the time of the latest follow-up were recorded.

**Results:**

Nine (60 %) of fifteen patients developed compartment syndrome secondary to trauma, four (4/15, 27 %) due to infection, and two (2/15, 13 %) due to intravenous infiltration. The average time from injury or hospital admission to fasciotomy was 31.8 h (range 2.9–136.3 h). In general, the functional outcome was excellent at the latest follow-up with thirteen (13/15, 87 %) patients having an excellent outcome. No cases of Volkmann’s ischemia were noted at the time of fasciotomy, even when performed as late as 5 days after injury.

**Conclusions:**

Compared to the general pediatric population, the diagnosis of compartment syndrome in infants and toddlers may be further delayed, i.e., >24 h after injury. Despite delays in diagnosis and time to treatment, the present study shows that outcomes in infants and toddlers remain favorable even when fasciotomy is performed 48–72 h after injury.

**Level of evidence:**

Case series, level IV.

## Introduction

Acute compartment syndrome (ACS) is a rare orthopedic emergency with serious and potentially devastating complications. Most available studies on ACS in children focus on older children [1–3]. The diagnosis of ACS in children can be quite challenging due to the variability of presentation, etiology and ability to communicate. Diagnosis can be even more difficult in very young children such as infants and toddlers aged <3 years. While the development of ACS in pediatric patients from supracondylar humerus and femur fractures has decreased, other etiologies have become more prevalent [2–5]. Lastly, children are known to be more resilient than adults and therefore have a better recovery potential even with a significant delay in diagnosis or treatment [6]. The purpose of this study was to evaluate the cause, diagnosis, treatment, and outcome of ACS in infants and toddlers aged <3 years.

## Materials and methods

After institutional review board approval, initial screening using ICD-9-CM codes for diagnosis of ACS and CPT codes for fasciotomy between April 1, 1999 and August 14, 2014 identified 35 patients aged <3 years at two level I pediatric trauma centers. Inclusion criteria were age <3 years at the time of injury and a clinical diagnosis of ACS requiring fasciotomy. Exclusion criteria were any patient who received prophylactic fasciotomy in the absence of clinical evidence of an acute or impending compartment syndrome, <3 weeks of follow-up, and age >3 years at the time of injury.

Retrospective electronic and physical chart reviews were performed to identify demographic information such as age at the time of injury, sex, height and weight, and body mass index or age-appropriate weight percentile using Center for Disease Control parameters [7, 8]. Presenting symptoms, associated injuries, anatomic location and mechanism of injury, compartment pressures (if available), time of injury (if available) and subsequent ACS diagnosis, time of fasciotomy, length of follow-up, and outcome at last follow-up appointment as defined by Flynn et al. [2] were also recorded. The time from injury to diagnosis, time from injury to fasciotomy, and time from diagnosis to fasciotomy were then calculated. The functional outcome at the time of latest outpatient follow-up was graded as ‘excellent’ (no loss of function or sensation), ‘fair’ (minor permanent change and/or the need for a minor assistive device such as an ankle–foot orthosis), or ‘poor’ (major loss of function and/or the need for a major assistive device to walk).

Patients were divided into three groups by etiology/cause—trauma-related (group A), infectious etiology (group B), and iatrogenic secondary to intravenous (IV) infiltration (group C).

## Results

**Fig. 1:**
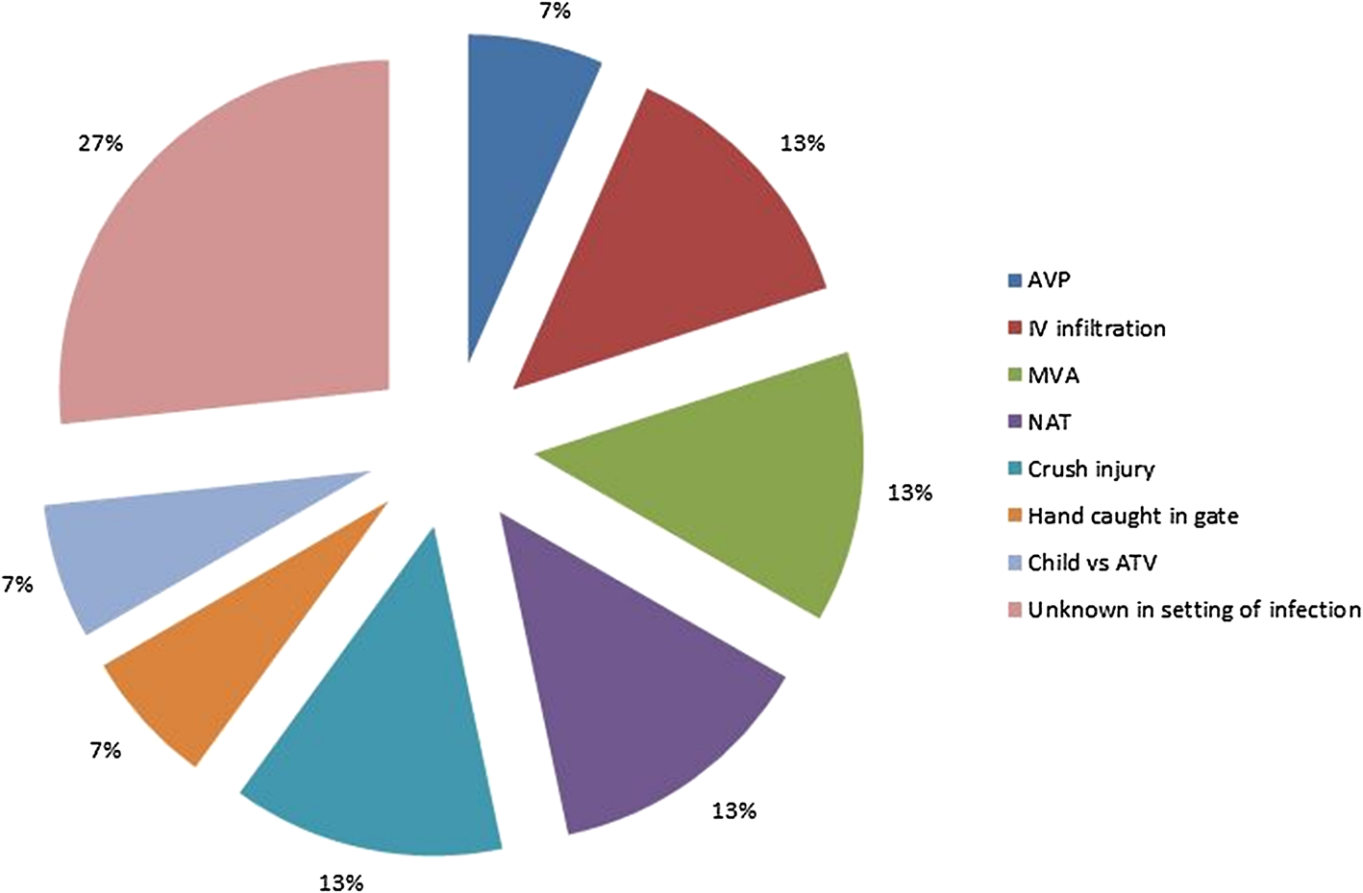
Breakdown of mechanism of injury resulting in compartment syndrome Reproduced with permission from the Children’s Orthopaedic Center, Los Angeles, USA

Fifteen patients (eight male, seven female) aged <3 years were diagnosed with ACS, and all underwent fasciotomy (Fig. 1). The average patient age was 12.1 months (range 6–32.6 months). The patients weighed an average of 10.2 kg at the time of presentation (range 5.6–17 kg) and averaged in the 47th percentile for weight (range <1 to >99th).

**Fig. 2:**
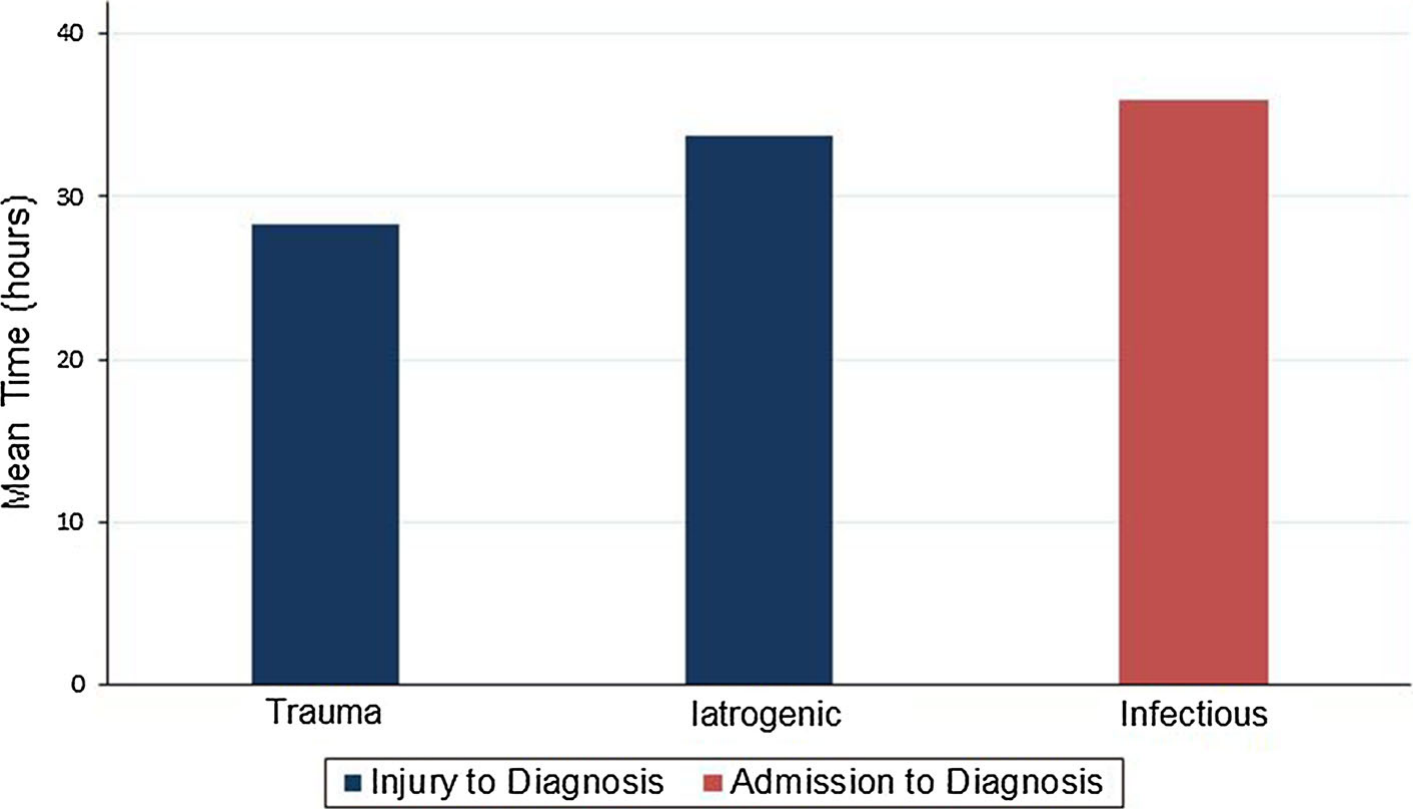
Time from injury or admission to diagnosis by type of injury Reproduced with permission from the Children’s Orthopaedic Center, Los Angeles, USA

Excluding ACS cases of infectious etiology, the overall average time from injury to compartment syndrome diagnosis was 29.4 h (range 3.0–134.4 h). The average time from injury to diagnosis for trauma-related cases (group A) was 28.3 h (range 3–134.4 h) and for IV infiltration-related cases (group C) cases was 33.8 h (range 3.3–64.8 h). The documented time of injury was not available in one trauma-related patient who was transferred from an outside hospital. As there was not a clear identifiable injury time in the infectious etiology group (group B), the time from admission to diagnosis was calculated and averaged 35.9 h (range 9.9–81 h). The overall average time from injury or hospital admission to diagnosis for all groups was 31.1 h (range 1.5–134.5 h). The overall average time from injury or hospital admission to surgery was 31.8 h (range 2.9–136.3 h) (Fig. 2).

The overall average time from diagnosis to treatment was 2.8 h (range 0–7.9 h). In the trauma-related group, the average time from diagnosis to treatment was 1.8 h (range 0–3.3 h), in the iatrogenic group 4.6 h (range 1.4–7.9 h), and in the infectious group 4.0 h (range 1.4–6.2).

Nine (60 %) of fifteen patients developed compartment syndrome secondary to trauma (Table 1). Four (44 %) of these nine patients were involved in a motor vehicle collision—two were struck by a motor vehicle and two were passengers in a car. The other trauma-related cases included crush injuries (three) and non-accidental trauma (two). All nine of the trauma-related cases sustained fractures—tibia and fibula (3), tibia (2), foot (1), humerus (1), and hand (2).

**Table 1:** Group A (trauma related): patient and injury characteristics, duration between events, and outcome

Case no.	Age (years)	Sex	Mechanism of injury	Compartment pressures recorded (Y/N; mmHg)	Location of injury (UE/LE)	Time from injury to diagnosis (h)	Time from diagnosis to surgery (h)	Outcome	Sequelae
10	0.56	M	NAT	N	R distal humerus (UE)	20	3.3	Excellent	None
1	0.54	F	NAT	N	L proximal tibia (LE)	33.2	1.67	Excellent	None
4	1.77	F	MVA	Y 88 (anterior) 67 (lateral) 62 (superficial posterior) 58 (deep posterior)	R tib/fib (LE)	13.8	1.32	Excellent	None
2	0.29	M	MVA	Y 51 (foot) 41 (ankle) 38 (leg)	L distal tibia (LE)	134.4	1.98	Excellent	None
5	2.43	F	Crush	Y 18 (medial) 50 (lateral) 55 (superficial & deep posterior) 8 (dorsal) 30 (plantar)	L 1st MT (LE)	3	2.17	Excellent	None
12	2.71	F	Crush	Y 20 (thenar) 11 (hypothenar)	R 2nd/3rd MC (UE)	5	1.9	Excellent	None
3	1.39	F	Crush	Y >40 (dorsal interosseous)	L 3rd/4th phalanx (UE)	10.5	0	Excellent	None
11	0.99	M	AVP	N	L tib/fib (LE)	–	2.15	Fair	Required 2 additional surgeries prior to closure; 26 degrees apex anterior deformity at latest follow-up
15	1.20	M	AVP	Y 55 (anterior /lateral) 45 (posterior)	L tib/fib (LE)	6.33	2.18	Excellent	None

*L* left, *R* right, *Y* yes, *N* No, *UE* upper extremity, *LE* lower extremity, *tib/fib* tibia and fibula fracture, *NAT* non-accidental trauma, *MVA* motor vehicle accident, *AVP* auto versus pedestrian, *MT* metatarsal, *MC* metacarpal, *FA* forearm

– Outside hospital transfer without documented time of injury

Infection was the underlying etiology in four (27 %) of fifteen cases of compartment syndrome. All four cases of infection-related compartment syndrome were caused by Gram-positive organisms [MRSA (2), MSSA (1), *Streptococcus pneumoniae* (1)] (Table 2). Two patients developed compartment syndrome in the setting of worsening sepsis and abscesses and soft tissue infections (cellulitis, myositis) of the lower extremities—one required fasciotomy of the thigh and ipsilateral lower leg, and the other required fasciotomy of the lower leg only. One patient developed compartment syndrome of the lower leg in the setting of lower leg infection plus septic ankle. The remaining infection-related case involved the upper extremity—a fasciotomy of the right hand with incision and drainage of a right subaponeurotic abscess.

**Table 2:** Group B (infectious): patient and injury characteristics, duration between events, and outcome

Case no.	Age (years)	Sex	Mechanism of injury	Compartment pressures recorded (Y/N; mmHg)	Location of injury (UE/LE)	Time from injury to diagnosis (h)	Time from diagnosis to surgery (h)	Outcome	Sequelae
9	0.70	F	Infection (MRSA)	N	R leg	–	5.4	Fair	Required 4 additional LE formal I&D prior to discharge; requires AFO; able to ambulate without pain at latest follow-up
14	0.75	M	Infection (MRSA)	Y >40 (all compartments)	R hand	–	2.98	Excellent	Required repeat I&D 4 days after fasciotomy
8	0.71	F	Infection (MSSA)	Y 61 (leg anterior) 83 (leg lateral) 28 (leg sup post) 22 (thigh anterior) 21 (thigh posterior)	R leg & R thigh	–	1.43	Excellent	Compartment syndrome with subsequent fasciotomies in both leg and thigh
7	0.90	M	Infection (pneumococcus 19A)	N	R ankle	–	6.22	Excellent	None

*N* no, *Y* yes, *R* right, *L* left, *MRSA* methicillin-resistant *Staphylococcus aureus*, *GPC* Gram-positive cocci (not speciated), *MSSA* methicillin-sensitive, *AFO* ankle−foot orthosis, *I&D* Irrigation and Debridement

Intravenous infiltration was responsible for two (13 %) of fifteen cases of compartment syndrome (Table 3). In this group, compartment syndrome developed in the forearm in one case and in the foot in another case.

**Table 3:** Group C (IV infiltration): patient and injury characteristics, duration between events, and outcome

Case no.	Age (years)	Sex	Mechanism of injury	Compartment pressures recorded (Y/N; mmHg)	Location of injury (UE/LE)	Time from injury to diagnosis (h)	Time from diagnosis to surgery (h)	Outcome	Sequelae
13	0.51	M	IV Infiltration	Y 68 (IO 1–2)	L foot	3.25	1.35	Excellent	None
6	1.39	M	IV Infiltration	Y 74 (volar)	L volar FA	64.4	7.87	Excellent	None

*FA* forearm, *UE* upper extremity, *LE* lower extremity, *Y* yes, *N* no, *L* left, *IV* intravenous

**Fig. 3:**
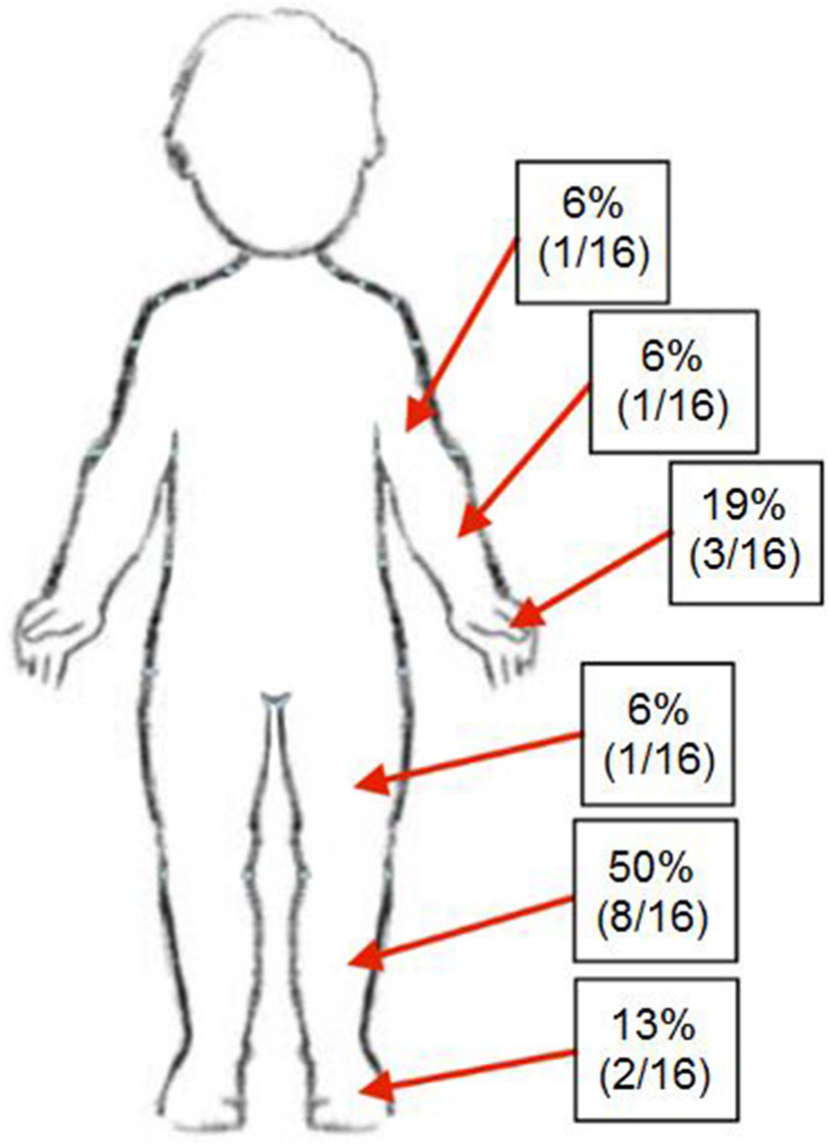
Breakdown of location of compartment syndrome Reproduced with permission from the Children’s Orthopaedic Center, Los Angeles, USA

The most common overall site of ACS and subsequent fasciotomy was the lower leg (8/16, 50 %) (Fig. 3). One of the eight patients also had concomitant fasciotomy for impending compartment syndrome of the ipsilateral thigh. The upper extremity was also commonly involved [hand (2); forearm (1); elbow (1)]. The foot was involved in two cases.

Pressure, or firm compartments (12/15, 80 %) and excess pain (12/15, 80 %) were the most common presenting symptoms of ACS (Table 4). Pallor (5/15, 33 %), paresthesia (1/15, 7 %), pulselessness (6/15, 40 %), and paralysis (4/15, 27 %) were also commonly present on examination. Six patients (5/15, 33 %) were reported to be agitated and two (2/15, 13 %) anxious. Compartment pressures were measured in ten of the fifteen patients (67 %), and recorded values were >40 mmHg in one or more compartments in all but one case. Documentation of anesthetic used during compartment measurements was not available in the majority of patients.

**Table 4:** Signs and symptoms leading to compartment syndrome diagnosis in all patients

Case no.	Excess pain	Pressure/firm compartment(s)	Pallor	Paresthesia	Pulseless	Paralysis	Agitation	Anxiety
1	Y	–	–	–	Y	–	–	–
2	Y	Y	Y	–	Y	–	Y	N
3	Y	Y	Y	–	–	–	–	–
4	Y	Y	–	–	Y	–	–	–
5	Y	Y	–	–	Y	–	–	Y
6	Y	–	Y	Y	Y	Y	–	–
7	Y	Y	N	N	N	N	Y	–
8	Y	Y	N	N	N	–	–	–
9	Y	–	N	N	N	N	Y	Y
10	–	Y	N	–	N	Y	–	–
11	Y	Y	Y	N	Y	Y	Y	–
12	Y	Y	–	N	N	Y	–	–
13	–	Y	Y	–	N	–	–	–
14	N	Y	N	N	N	N	Y	–
15	Y	Y	N	N	–	–	–	–

*Y* yes, *N* no, – not reported

Average duration of follow-up was 10.2 months (range 0.90–26.5 months). Thirteen (87 %) of the fifteen patients had an excellent outcome at the latest follow-up. Of the two patients with a fair outcome, one patient with compartment syndrome of infectious etiology (group B) developed ankle valgus instability due to loss of the fibular buttress and required brace treatment (ankle−foot orthosis). The second, who was struck by a motor vehicle (group A), was lost to follow-up after 2.9 months and was noted to have a 26-degree apex anterior malangulation of the distal tibia. No significant difference in outcome was found between patients treated <24 h (7 patients) after injury compared to ≥24 h (3 patients) after injury (*p* = 0.99).

## Discussion

The challenges of diagnosing compartment syndrome in the general pediatric population have been well-established and documented in the literature. Delayed diagnosis of compartment syndrome in children is not uncommon. The average time from injury to diagnosis of compartment syndrome was reported to be 18.2 h in a retrospective series of 43 children who developed ACS of the lower leg secondary to trauma [2]. A retrospective series of 23 children reported an average time from upper extremity injury to emergency room presentation of 9.5 h and from presentation to diagnosis of 16 h [9]. This series of compartment syndrome in infants and toddlers (<3 years of age) reveals the significant diagnostic challenge presented by this age group, with an average delay of 31.1 h.

The reasons for delayed diagnosis of compartment syndrome in this younger population are multi-factorial. For example, longer elapsed time between initial injury and peak compartment pressures have been reported in the pediatric setting [2]. Transfer times may also certainly add to the delay. This younger population of infants and toddlers is often non-verbal, easily irritable (apprehensive, crying), and too young to cooperate; all factors in making a clinical diagnosis of compartment syndrome very difficult. The fact that pain was the most common presenting symptom (12 of 15 reported) and firm compartments the most common physical finding (12 of 15) in this series highlights the challenge of making a timely diagnosis. Pain is non-specific and an assessment of compartment firmness (or pressure) by manual palpation has been shown to be unreliable, with a sensitivity of only 54 % for compartment syndrome [10]. Because of the difficulty in making (or excluding) a diagnosis of compartment syndrome clinically, compartment pressure measurements may be needed. Compartment pressures, however, are difficult to measure in an awake infant or toddler and often require conscious sedation or anesthesia.

This delay in diagnosis highlights the need for improved methods to diagnose compartment syndrome in at-risk populations. Near-infrared spectroscopy has been proposed as a noninvasive, reproducible, and accurate method to measure tissue oxygenation and diagnose compartment syndrome [11]. Tissue oxygenation levels were reported to be significantly lower in patients with compartment syndrome compared to a matched control group with lower extremity injuries and no compartment syndrome [11]. Limitations due to the effects of skin pigmentation and subcutaneous fat, however, have been cited, and future studies to clarify its clinical application in ACS are needed. Radiofrequency identification implants are a newer technology that also has the potential to diagnose compartment syndrome accurately and reproducibly. These simple, miniature implantable devices can measure pressure and temperature fluctuations in the limb compartments of patients at risk for developing ACS [12].

The importance of timely diagnosis and treatment is to avoid sequelae related to ischemic insult, i.e., tissue necrosis, neurovascular compromise, and permanent functional deficits. Late diagnosis can increase the risk for severe complications, including infection, neurologic injury, need for amputation, and death. Especially in the adult population, concerns about increased risk of muscle necrosis and infection have led to some recommendations not to perform a fasciotomy 24 h after the onset of symptoms [13–15]. However, this series of pre-school-aged children (aged <3 years) shows that the outcome can be favorable even when surgical decompression (fasciotomy) is performed 72 h after injury. In fact, there were no cases of Volkmann’s ischemia at the time of fasciotomy, even when performed as late as 5 days after injury. Similarly, good results have also been reported in the general pediatric population even when fasciotomy is performed late. In one series of fourteen patients, all had an excellent outcome even when fasciotomy was performed for acute compartment syndrome of the leg secondary to tibial and/or fibular fractures as late as 72 h after the injury [2].

The clinical presentations of compartment syndrome in infants and toddlers are varied. Consistent with the literature, trauma to the lower leg was the most common cause in this series (8 of 16 cases) [16]. Non-accidental trauma led to two cases. Other etiologies in our series included IV infiltration and infection. Different theories have been proposed to explain the increased risk of IV infiltration in the pediatric population—(1) increased velocity of fluid through a smaller catheter tip; and (2) increased fragility of pediatric veins [17]. In a 25-year review of the literature, Paley et al. reported nine cases of compartment syndrome secondary to infection [18]. Pyomyositis, necrotizing fasciitis and streptococcal toxic shock syndrome are infectious causes known to cause compartment syndrome [19].

The risk of tissue necrosis and subsequent neurovascular compromise leading to permanent functional deficits differs depending on the cause/etiology (trauma, infection, IV infiltration) and involved pathophysiology. In trauma-related compartment syndrome, sustained increased pressure within an osseofascial compartment/space from tissue edema and/or hemorrhage can compromise circulation and lead to tissue ischemia, cellular anoxia, and ultimately tissue death. This may also be exacerbated by positioning and cast placement as in Volkmann’s contracture after spica casting of femur fractures [20]. The pathologic mechanism by which infection leads to compartment syndrome is not entirely known but likely involves an inflammatory response in the tissues resulting in endothelial injury and increased vascular permeability [14]. Lastly, the pathophysiology in compartment syndrome due to IV infiltration is different because of the subcutaneous space involvement, expansile skin, and lack of rigid boundaries [12].

There are several limitations in our study. First, because of its retrospective design, the study was limited by incomplete medical records. For example, we did not have compartment pressures and systemic blood pressures for all patients. An important point, however, is that the diagnosis of compartment syndrome can be made on clinical grounds alone [21]. Measuring compartment pressures can sometimes be helpful if the clinical picture is questionable or equivocal. Inconsistent availability of data also prevented us from analyzing analgesic requirements and their utility as an indicator of ACS as reported by Hosseinzadeh and Talwalkar [22]. Second, while it is useful to study the ‘time from injury to diagnosis and treatment’ of compartment syndrome, it is probably more relevant to study the ‘time from increased pressure to diagnosis and treatment’. However, as the exact time of increased pressure is virtually impossible to determine, we used ‘time from injury’ as an adjunct marker. Next, due to the rarity of compartment syndrome in infants and toddlers, the sample size during the 15-year study period was small. We chose therefore to include and review cases of ACS due to multiple etiologies and recognize that the natural history and treatment protocols may differ depending on the underlying etiology. To date, this series represents the largest series investigating ACS in the very young population of infants and toddlers. One final limitation was the short amount of follow-up time. As the majority of patients experience a rapid and excellent recovery following treatment for ACS, it is common for patients not to return for long-term follow-up evaluations. Although a minimum 3-week follow-up should be a reasonable threshold for recognizing early complications of compartment syndrome, it is possible that complications manifested later and were missed.

In conclusion, the present study shows that compared to the general pediatric population, the diagnosis of compartment syndrome in infants and toddlers may be further delayed, i.e., >24 h after injury. The diagnosis of compartment syndrome in this younger population is an extreme challenge, and heightened awareness is needed. Trauma-induced fractures appear to be the most common cause of compartment syndrome in infants and toddlers; however, it is necessary to be suspicious for compartment syndrome in the setting of infection, IV infiltration, and non-accidental trauma. Prompt fasciotomy continues to be the recommended treatment (when possible), but the present study shows that outcomes in infants and toddlers remain favorable even when fasciotomy is performed 48–72 h after injury.
